# The Relevance of GIRK Channels in Heart Function

**DOI:** 10.3390/membranes12111119

**Published:** 2022-11-09

**Authors:** Ana Campos-Ríos, Lola Rueda-Ruzafa, José Antonio Lamas

**Affiliations:** 1 CINBIO, Laboratory of Neuroscience, University of Vigo, 36310 Vigo, Spain; 2 Laboratory of Neuroscience, Galicia Sur Health Research Institute (IISGS), 15706 Vigo, Spain; 3 Department of Nursing Science, Physiotherapy and Medicine, Faculty of Health Sciences, University of Almeria, 04120 Almeria, Spain

**Keywords:** heart, GIRK channel, IK, ACh, G-protein-coupled receptor

## Abstract

Among the large number of potassium-channel families implicated in the control of neuronal excitability, G-protein-gated inwardly rectifying potassium channels (GIRK/Kir3) have been found to be a main factor in heart control. These channels are activated following the modulation of G-protein-coupled receptors and, although they have been implicated in different neurological diseases in both human and animal studies of the central nervous system, the therapeutic potential of different subtypes of these channel families in cardiac conditions has remained untapped. As they have emerged as a promising potential tool to treat a variety of conditions that disrupt neuronal homeostasis, many studies have started to focus on these channels as mediators of cardiac dynamics, thus leading to research into their implication in cardiovascular conditions. Our aim is to review the latest advances in GIRK modulation in the heart and their role in the cardiovascular system.

## 1. Introduction

Among the variety of potassium channels involved in cardiac electrical activity [1,2,3], G-protein-gated inwardly rectifying potassium (GIRK) channels, also known as Kir3 channels, appear as contributors to resting membrane potential [4,5]. They are members of a subfamily of inwardly rectifying channels (Kir1–Kir7), that preferentially conduct larger inward currents at negative voltages to the K+ equilibrium potential than outward currents at positive voltages, a phenomenon known as inward rectification [4,5].

GIRK channels are activated in response to neurotransmitters such as γ-aminobutyric acid, serotonin, dopamine, or opioids, interacting with G-protein-coupled receptors (GPCRs) [6]. GPCRs constitute a large family of seven-transmembrane (7TM) receptors encoded by over 800 genes [7]. Their signalization via heterotrimeric G proteins (composed by α and βγ subunits) regulates several physiological processes and serves as a target of many drugs. Once activated, intracellular Gα and Gβγ protein effectors are released and they trigger a cascade that generates several molecular activities, such as the activation of GIRK channels in both the heart and the brain [7,8]. The βγ-subunit of the heterotrimeric G-protein complex Gαβγ is believed to be responsible for activating these channels [9,10,11,12], which in turn hyperpolarize the cell membrane with a subsequent decrement in excitability [5].

Regarding heart regulation, sympathetic and parasympathetic branches of the autonomic nervous system (ANS) tightly control heart rate by stimulating different GPCRs, responsible for activating ion channels that modify the electrical properties of cardiac pacemaker cells [13]. Parasympathetic fibers travelling over the vagus nerve mainly target in the sinoatrial (SA) node, slowing its pacemaker cells by hyperpolarizing them, and atrioventricular (AV) node, diminishing its conduction, thus leading to a decrease in heart rate. By contrast, the sympathetic nervous system has a more ventricular distribution and predominantly increases the heart rate and the myocardial contractility [14].

In this way, GIRK channels are considered largely responsible for controlling heart neuronal excitability, so it is not hard to imagine that imbalances in the function of these channels can lead to disturbances in heart rhythm [15] and thus, to many cardiac disorders [16,17,18,19,20]. We therefore review the role of GIRK channels in the functioning of the heart and their involvement in its physiology and pathophysiology.

## 2. Structure and Signaling

As we have mentioned before, the heart is thoroughly controlled by the ANS. The diminished heart rate mediated by vagus nerve stimulation and chemical synaptic transmission was first shown by Otto Loewi in 1921 [21,22]. In this work, a chemical substance called Vagusstoff was found. This stimulated the vagus nerve causing a decrease in heart rate. This substance was revealed to be acetylcholine (ACh) [21]. The vagus nerve releases ACh activating M2 muscarinic receptors (M2R) in the SA node cells, which promotes the formation of the GPCR–Gα(GDP)βγ complex and subsequently, the exchange of GDP to GTP occurs at the Gα subunit. Then, dissociation of Gα(GTP) and Gβγ subunits takes place [14,23]. The Gβγ subunit specifically derived from Gi/o [24] binds to GIRK channels, activating them and generating an inward potassium current [15,25]. The opening of GIRK channels leads to the hyperpolarization of the cell membrane and consequently reduces the heart rate [13,23]. Even though β-adrenergic receptors of the sympathetic system liberate Gβγ when activated, this is not sufficient for activation of GIRK currents, except in heterologous systems or when atrial myocytes overexpress β1-adrenergic receptors [26]. Touhara and MacKinnon (2018) found that M2Rs catalyzes Gβγ subunit release at higher rates and generates higher GIRK protein concentrations [23].

Because ACh is the component that triggers the mentioned G-protein-dependent pathway, GIRK channels are also known as KACh channels, and they were first described in frog atrial myocytes [6]. The current flowing through them, called the muscarinic-receptor-activated current (IKACh), was reported as one of the main Kir channel currents in cardiac tissue [12]. These channels are composed of four subunits consisting of two α-transmembrane helices (M1 and M2), a pore-forming p helix that acts as a selective filter for potassium ions and an intracellular C- and N-termini [27]. Four subunits (GIRK 1 to 4) that assemble into homo- and heterotetrameric channels have been cloned in mammalian cells [25]. It has been shown that heteromultimerization among distinct subunits may lead to different functionally expressed GIRK channels with distinct G-protein-activated currents [28], although less is known about the physiological relevance of this matter.

### 2.1. GIRK1

GIRK1 (Kir3.1) is encoded by the KCNJ3 gene and is located in chromosome 2 in humans [29]. It was first cloned in 1993 by Kubo and colleagues and found to be expressed both in the heart (mostly atria and SA node) and brain [30]. Although GIRK1 functional homotetramers have not been reported [31], when coassembled with one of the other three subunits, GIRK1 clearly enhances the channel activity [32]. A Q404-specific residue in the carboxyl-terminal and three residues in the pore-forming loop are determinant characteristics of GIRK1, increasing the open probability and channel conductance of the formed heterotetramer [32]. Along with Kir3.4 (KCNJ5), it creates the heterotetrameric KACh channel in the atria, the major and most important cardiac configuration [33]. This multimeric form plays a fundamental role in regulating cardiac rhythm and it is known that, in the presence of cholesterol, its open probability increases [34]. In addition to the heart, Mett et al. [31] clarified the importance of GIRK1 heteromeric forms in hippocampal brain function, such as synaptic plasticity and memory, by using a knock-in mouse model [31]. Other studies have shown its role in the correct neuronal function in the cerebellum and thalamocortical regions [35,36,37]. Related to certain conditions, high gene expression has been directly linked to breast cancer [38,39], while its downregulation in the prefrontal cortex is associated with schizophrenia [40].

### 2.2. GIRK2

GIRK2 or Kir3.2 channels (KCNJ6 gene) are major players in the nervous system. Together with Kir3.1, these channels are known to be the most abundant subunits in the mammalian brain. Although they can assemble and form functional identical subunit homomers, GIRK2/2 seems to be less sensitive to activation than heterotetramers conformed with GIRK1 subunits [41]. As mentioned, the GIRK1/GIRK2 channel has been found to be the most abundant heteromultimer in the brain and cerebellum [42] and it controls neuronal excitability by lengthening the interval between action potentials, creating a slow inhibitory post-synaptic current in most of the brain regions [43]. It has also been reported than N-methyl-D-aspartate (NMDA) receptor activation might lead to increase in GIRK1/2 trafficking to the surface in response to different neuromodulators [44]. GIRK2 can also form functional homomers in dopaminergic neurons of the substantia nigra [45,46]. They display a “burst kinetic”, which means they rapidly switch between open and closed states when activated [47]. A special feature of these channels is that they present a specific aminoacidic domain on the surface that creates the binding site of the Gβγ subunit. It has been shown that atomic and electrostatic interactions might trigger a pre-open state, where phospatydilinositol-4,5-biphosphate (PIP2) and Na^+^ ions enhance the activation, creating a multi-ligand modulation of the channel gating [47].

### 2.3. GIRK3

GIRK3 (Kir3.3) is encoded by the KCNJ9 gene in humans and is widely distributed in the rodent brain [48,49,50]. It cannot form functional homotetramers [41], but it is capable of assembling with the other members of the GIRK subfamily. When coexpressed with GIRK1, enhanced evoked currents appear [28]. Along with GIRK2 channels, they are exclusive in dopaminergic neurons and the ventral tegmental area [41]. It has also been reported that there is a clear relationship between GIRK3 and cellular and behavioral effects of ethanol [51], as well as a link between these channels and dopaminergic sensitivity to abusive drugs such as cocaine [52].

### 2.4. GIRK4

KCNJ5 is the gene encoding Kir3.4, also known as GIRK4 [53]. Together with Kir3.1, it forms heterotetramers, creating the mentioned complex responsible for IKACh in the heart [33]. High protein expression of this form was found in the atria [54] and in the paraventricular nucleus of the hypothalamus, a region in charge of cardiac vagal neuron regulation [55,56]. It does not seem to appear in many regions in the brain or be important in neuronal regulation, apart from spatial learning and memory in mice [56]. A study has shown that alterations in two single-nucleotide polymorphisms (SNPs) of this gene are related to a higher probability of suffering atrial fibrillation under 40 years old [19]. It has also been shown that aldosteronism, a condition that occurs when adrenal glands segregate high aldosterone levels, may occur because of mutations in the KCNJ5 gene in some patients [57]. In severe cases of aldosteronism, two mutations make these potassium channels permeable to Na^+^ [58].

## 3. GIRK Pharmacology

**Figure 1:**
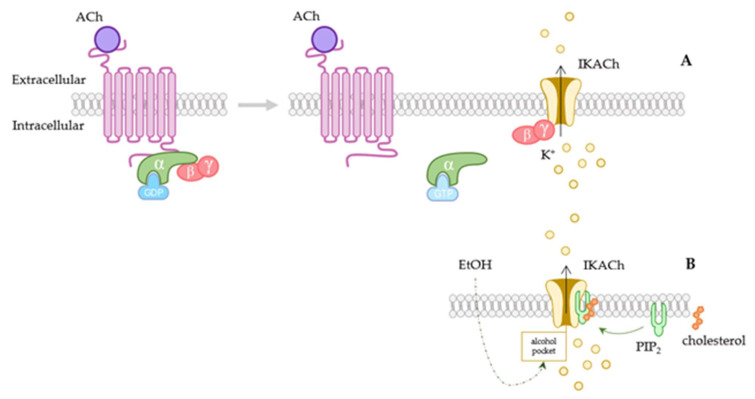
Activation of GIRK channels. (**A**) Scheme of the GIRK channel G-protein-coupled receptor-dependent activation pathway. When acetylcholine (ACh) binds to the receptor, it causes the dissociation of Gα(GTP) and Gβγ subunits. Gβγ intracellularly attaches to the channel, provoking its opening and muscarinic-receptor-activated current (IKACh) activation. In the upper scheme both atrial and ventricular cardiac potential are shown, together with the contribution of IKACh current driven through GIRK1/4 channel (purple trace). These channels contribute to the resting membrane-potential maintenance and to slowdown excitability. (**B**) G-protein-independent pathways, such as the ones caused by alcohol or by cholesterol have also been reported. These are both PIP2-dependent. Ach: acetylcholine; IKACh: muscarinic-receptor-activated current; PIP2: phosphatidylinositol-4,5-biphosphate.

In addition to the G-protein signaling pathway, recent studies revealed that other substances can modulate GIRK channels (G-protein-dependent and -independent pathways are shown in Figure 1). It was observed that ethanol activates GIRK channels in a G-protein-independent manner both in vitro and in vivo [59,60]. Its activation is due to the existence of an alcohol-binding hydrophobic pocket first reported in GIRK2 [61,62]. It was also described as a GIRK1/2 channel activator called GiGA1 (G-protein-independent activator 1), that modulates the channel in native and heterologous systems [63], and ML297, a potent activator with a high efficacy and preference for GIRK1/2 combination [64]. Furthermore, GIRK channels are typically G-protein-dependent and activated by inhibitory neurotransmitters such as ACh [18] or B-type G-protein-coupled γ-aminobutyric acid receptors (GABABR), opioids, or serotonin [6,65]. Similarly to what happens in the brain, cholesterol is known to increase the open probability of GIRK cardiac heterodimers (GIRK1–GIRK4) [34]. By using the GIRK2 gene reconstituted into liposomes, it has been shown that both alcohol and cholesterol need the presence of PIP2 to directly activate the channels [66,67,68]. It also has been found that the antiparasitic drug ivermectin activates GIRK channels in a PIP2-dependent manner [69]. The mentioned studies show a possible relevant role of PIP2 in GIRK channel modulation, which is essential in maintaining channel activity [70]. These channels can present different configurations, and PIP2 might cause structural changes that help the Gβγ subunit to reach its binding side [71]. More research focusing on these changes in configuration might help to understand G-protein-independent activation of these channels. Furthermore, they are negative modulated by many different antidepressants, such as fluoxetine [72] or paroxetine [73] or by the norepinephrine-reuptake inhibitors atomoxetine and reboxetine [74]. For their part, regulators of G-protein-signaling (RSG) proteins play a fundamental role controlling GIRK channel activity. These proteins regulate GIRK channels in an allosteric manner by forming macromolecular complexes [75] and regulating channel-gating behavior [76]. For example, RGS4 attenuates sinus rhythm of the SA node by diminishing GIRK channel activity [77]. For its part, RSG6 also plays a fundamental role in weakening parasympathetic activation and preventing bradycardia [78,79], and RGS6-deficient myocytes cause a GIRK channel desensitization and higher activation deactivation rate [78]. Generally, it has been found that G-protein-independent modulation might play a more relevant role than first thought. Further information about GIRK pharmacological regulation can be found in the most recent review by Jeremic et al. (2021) [18].

The implication of these channels in GPCRs modulatory pathways in such a diverse manner makes GIRK channels an interesting partaker in heart-excitability control.

## 4. GIRK in the Heart

Broadly, the four subunits GIRK1 to 4 are widely expressed in mammals [5,8]. GIRK1-3 subunits have been found prominently in the brain, while GIRK4 is mainly located in cardiac tissue [5], forming the main GIRK heterotetramer in the heart along with GIRK1.

Regarding rodents, elevated mRNA levels of GIRK1, GIRK4, and M2R appear in mice atria and less strongly in the ventricle [80]. The GIRK1 channel is also strongly expressed in rat, ferret, and guinea-pig atria, but not ventricles [54,81]. Its mRNA expression was detected by PCR in both ferret atria and ventricles, as well as in dog ventricles. Although Kir3.1 and Kir3.4 protein expression was low in ferret ventricle-tissue sections and isolated cells, its expression was high in the atria [82]. Similarly, GIRK4 was found in the intercalated discs and in the external sarcolemma of the right atrium, whereas it was only shown in the left-ventricle intercalated discs by image experiments performed in rats [83]. Its expression was also determined by immunofluorescence in rat atria and scarce in ventricles [81]. In humans, GIRK4 presence in left-ventricular epicardial and human endocardial sections, specifically in the intercalated discs and slightly less in the t-tubules, was demonstrated; however, GIRK4 was not detected in mid-myocardial sections in the left ventricle [83]. All these data are summarized in Table 1. It seems that the GIRK1/4 complex predominantly appears in supraventricular SA node cells and myocytes, where it might have a most relevant role in cardiac physiology.

As mentioned before, the activation of IKACh leads to a membrane potential hyperpolarization, a slowdown of the spontaneous firing rate and, thus, a delay in atrioventricular conduction [18]. These currents mainly appear in the atrial area, and they are implicated in the cardiac action potential repolarization [84]. It is worth mentioning that, despite it has been suggested that cholinergic innervation (vagal efferents) may not have a significant role in ventricular function [85,86], it has also been found that the activation of GIRK channels in ventricular myocytes might cause higher density currents than previously reported [87], although its physiological relevance has not been clearly elucidated. More comparative research on their function in atria versus ventricles might help to clarify their significance in ventricular function.

Even so, there is no doubt that vagal stimulation has a crucial parasympathetic influence on cardiovascular physiology, that decreases heart rate through the main action of GIRK channels. Thus, it is not hard to imagine that aberrant variations in GIRK activity due to functional mutations might perpetuate atrial fibrillation and other cardiac pathogenesis. Several investigations suggest an important role of GIRK channels in cardiovascular pathophysiology (Table 2) and many studies in animal models has been developed in recent decades. In this regard, a direct impact of vagal stimulation on heart rate and the atrial activity of GIRK channels has been observed. Heart rate dynamics and control of parasympathetic regulation was affected after ablation of GIRK1 and 4 genes and subsequent vagal stimulation in mice [55]. Specifically, it was observed that IKACh mediated half of the negative chronotropic effects of vagal stimulation on heart rate, disrupting GIRK4 gene coding [15]. Similarly, the use of carbachol, a cholinergic agonist, failed to activate the IKACh and thus induced atrial fibrillation in mice lacking the GIRK4 gene, suggesting its importance in atrial heterotetramer physiology [88].

As described by Lee and colleagues (2018) [55], GIRK channels are the main contributors to heart rate via vagal nerve stimulation, specifically in the atria. Abnormalities in pacemaker conduction might lead to cardiac arrhythmias and heart failure. A study found that both GIRK4 and adenosine 1 receptor mRNA expression was augmented in isolated SA node myocytes of a heart-failure canine model. This generated a reduction in the hyperpolarization rate in the presence of adenosine, proving a possible protective role of adenosine-1 receptor trough the blockade of GIRK channels, which might prevent SA node dysfunction and subsequent arrhythmia [89]. Another study of canine atrial tachycardia showed a possible Kir3 channel role in cardioprotective effects against arrhythmia. As these channels contribute to action potential shortening in cardiomyocytes, its selective blockade by their antagonist tertiapin-Q suppressed atrial arrhythmias without affecting ventricle conductance [90]. By using tertiapin-Q, another work showed the implication of GIRK current cardiomyocytes from the pulmonary veins [91], with a well-known role in atrial fibrillation initiation and maintenance [92,93].

Regarding human studies, disruption of GIRK and especially GIRK4 has also been linked to cardiovascular alterations such as hypertension or atrial fibrillation in humans. Thus, a heterozygous mutation of the Kir3.4 (Kir3.4-Gly387Arg) was identified in all the affected members of a family with autosomal-dominant congenital long QT syndrome (LQTS). This mutation revealed a loss of function resulting from a reduced plasma membrane expression and a subsequent decrement in the current amplitude and a prolongation of cell repolarization, which, among other symptoms, led to recurrent syncopal episodes [20]. Other research showed that variations in heart rate could stem from genetic variants of proteins involved in Gβγ sinoatrial signaling induced by the GIRK channels [94]. IKACh has also been found constitutively active in chronic atrial fibrillation patients, resulting from an abnormal phosphorylation by protein kinase C [95,96].

Furthermore, a left-to-right atrium gradient GIRK current was found to contribute to paroxysmal atrial fibrillation [97], thus leading to the possibility of a selective-localization approach to treat specific cardiac diseases. Regarding adenosine-induced atrial fibrillation, a study with 37 human hearts showed that GIRK4 channel blockade might prevent the shortening of action potential and subsequent atrial fibrillation, as these channels, along with adenosine 1 receptor, were overexpressed in those areas with greatest adenosine-induced action potential shortening [98]. By using shRNA adenovirus against GIRK4, Liu et al. achieved an efficient silencing of the channel in human atrial myocytes [99]. This could be a potential way to control arrhythmia mediated by GIRK overactivation. It is worth mentioning sinus-node dysfunction (SND), a disorder characterized by poor conduction between the SA node and the atria that generates an abnormal pacing rhythm [100]; however, the data published to date are contradictory. On the one hand, after sequencing the KCNJ3 and KCNJ5 genes, which encode the main subunits of KACh channels in the heart, from almost 50 patients, no mutation was found that could link these channels to SND [100]. Nevertheless, a few years later, a study found that a gain-of-function mutation in the human GIRK4 gene causes familial SND by enhancing the activity of these channels in pacemaker cells and resulting in a sustained reduction in heart rate [101]. Other research hypothesized that the G-protein-signaling regulator RGS4, highly expressed in SA node pacemaker cells, modulates GIRK channels in SA node myocytes; its loss of function might lead to atrial fibrillation and SND through to GIRK channel overfunction [102].

**Table 2:** Effect of GIRK1/4 modifications in cardiac physiology.

Modification	Species	Effect in Cardiac Physiology	Reference
GIRK1 gene ablation GIRK4 gene ablation	Mouse	Loss of parasympathetic regulation Loss of heart rate dynamics	[55]
GIRK4 gene disruption	Mouse	IKACh effect in heart rate	[15]
GIRK4 knockout mice	Mouse	Atrial fibrillation	[88]
↑ GIRK4 mRNA in sinoatrial myocytes	Dog	Heart failure	[89]
GIRK blockade	Dog	Suppression of atrial arrythmias	[90]
GIRK genetic variations	Human	Βγ-signaling pathway variations Heart-rate variations	[94]
GIRK4 mutation	Human	Familiar LQTS	[20]
IKACh constitutive activation	Human	Chronic atrial fibrillation	[95,96]
IKACh gradient current	Human	Paroxysmal atrial fibrillation	[97]
GIRK4 overexpression	Human	Protective against adenosine-induced atrial fibrillation	[98]
GIRK4 silencing	Human	Arrhythmia-control mechanism	[99]
GIRK4 gain-of-function	Human	Familial SND	[101]
GIRK overfunction	Human	Atrial fibrillation and SND	[102]

IKACh: muscarinic receptor-activated current; SND: sinus-node dysfunction; LQTS: long QT syndrome augmented.

**Figure 2:**
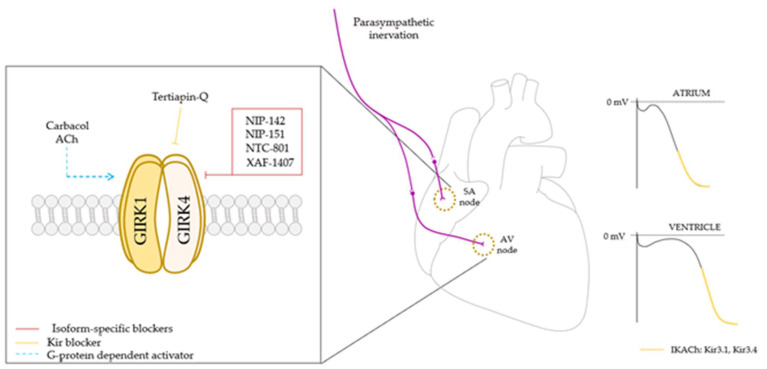
GIRK1/4 heterotetrameric channel controls heart rate via parasympathetic innervation. GIRK1/4 channel activation leads to a membrane potential hyperpolarization and a slowdown in firing rate (right), and subsequently a decrease in heart rate, being more relevant in the atria. As it is a promising target to treat arrhythmias and atrial fibrillation, and despite Kir channel blockers are already known to be capable of regulating channel activity, in the last few years several isoform specific blockers have been developed in order to achieve a more detailed control of the channel activity and to act as antiarrhythmic potential drugs. SA node: sinoatrial node; AV node: atrioventricular node; ACh: acetylcholine.

Altogether, these studies show GIRK channels to be a promising tool in protecting the heart from atrial fibrillation and arrhythmias (Figure 2). In recent years, many studies have developed new selective blockers targeting the GIRK1/4 heart isoform in order to modulate the changes in action potential duration in the atria [103]. As GIRK channel modulation seems to be a good approach to treat atrial fibrillation and conduction abnormalities, some compounds were tested in recent years to achieve this objective [103]. NIP-142 and NIP-151, small selective inhibitors, provided a vagal nerve silencing without affecting the electrophysiology of the heart [104,105]. Another compound called XAF-1407 was shown to have antiarrhythmic properties through the inhibition of IKACh without side effects [106]. NTC-801 is also an effective antiarrhythmic GIRK channel blocker [107]. With the actual knowledge about the differential localization and modulation of cardiac GIRK1/4, it seems that this channel is a promising tool in abnormal arrhythmogenesis regulation and in electrical activity control.

## 5. Conclusions

The expression of GIRK channels in the heart provides a control point in cardiac physiology. Many of the findings to date have revealed that IKACh flowing through GIRK channels would strongly contribute to cardiac electrical excitability and thus to cardiac pathophysiology. The incidence and prevalence of these diseases are increasingly globally, and although more research is needed to elucidate its role in pathologies such as atrial fibrillation, we have observed that an extensive bibliography highlights the contribution of the GIRK1/4 heterotetrameric isoform in heart rate regulation and electrical activity control, and also that this isoform has been linked to cardiac alterations. This manifests the importance of focusing future research on the synthesis of new potential drugs to specifically target this channel and to find an approach to selectively direct these newly developed treatments to localized affected areas in specific cardiac diseases.

## Figures and Tables

**Table 1:** Expression of GIRK channels in the heart of different species.

Subunit	Species	Location in the Heart	Expression Determination	Reference
GIRK1 GIRK4	Mouse	Atria Ventricles	mRNA expression	[80]
GIRK1	Rat	Atria	Protein expression	[54,81]
GIRK1 GIRK4	Guinea pig	Atria Ventricles	Protein expression	[54]
GIRK4	Rat	Right atrium (intercalated discs and sarcolemma) Left ventricle (intercalated discs)		[83]
GIRK1 GIRK4	Ferret	Atria Ventricles	mRNA expression Protein expression	[81]
GIRK1	Dog	Atria Ventricles	mRNA expression	[82]
GIRK4	Human	Left ventricle (intercalated discs and t-tubules)	Protein expression	[83]

## Data Availability

Not applicable.
